# Dietary fat and fatty acid intake and epithelial ovarian cancer risk: evidence from epidemiological studies

**DOI:** 10.18632/oncotarget.5525

**Published:** 2015-10-26

**Authors:** Rui Hou, Qi-Jun Wu, Ting-Ting Gong, Luo Jiang

**Affiliations:** ^1^ Department of Obstetrics and Gynecology, Shengjing Hospital of China Medical University, Shenyang, China; ^2^ Department of Clinical Epidemiology, Shengjing Hospital of China Medical University, Shenyang, China; ^3^ Department of Ultrasound, Shengjing Hospital of China Medical University, Shenyang, China

**Keywords:** diet, fat, fatty acid, meta-analysis, ovarian cancer

## Abstract

The associations between dietary fat and fatty acid (FA) intakes and epithelial ovarian cancer (EOC) risk have been inconsistent in previous studies. We conducted a meta-analysis of epidemiological studies to evaluate these associations. We identified relevant studies by searching PubMed, EMBASE, and Web of Science databases. We used random-effects models to estimate summary relative risks (RRs) and 95% confidence intervals (CIs). Overall, the search yielded 20 studies (1 pooled analysis of 12 cohort studies, 5 cohorts, and 14 case-control studies). The summary RR for EOC for the highest versus lowest categories of total dietary fat intake was 1.12 (95%CI= 0.95–1.33; *I*^2^ = 77.4%; *n* = 14). The RRs were not significant when fats were divided into plant-based fats (RR = 0.93, 95%CI = 0.77–1.13; *n* = 6), animal-based fats (RR = 1.15, 95%CI = 0.95–1.39; *n* = 8), dairy-based fats (RR = 1.02, 95%CI = 0.88–1.18; *n* = 3), saturated FAs (RR = 1.04, 95%CI = 0.93–1.17; *n* = 12), monounsaturated FAs (RR = 0.98, 95%CI = 0.84–1.13; *n* = 10), polyunsaturated FAs (RR = 0.96, 95%CI = 0.81–1.12; *n* = 10), and trans-unsaturated FAs (RR = 1.15, 95%CI = 0.98–1.36; *n* = 3). Similar non-significant results were also observed in most of the subgroup and sensitivity analyses. The findings of this meta-analysis suggest a lack of evidence for associations between dietary fat and FA intakes and EOC risk. Further analyses should be conducted to assess the associations with other types of fat, and the results should be stratified by tumor invasiveness and EOC histology.

## INTRODUCTION

Ovarian cancer is the most lethal gynecologic cancer worldwide, accounting for approximately 240,000 cases and 150,000 deaths in 2012 [1]. Approximately 90% of invasive ovarian cancers are classified as epithelial ovarian cancer (EOC), which arises from the surface epithelium of the ovary. Nearly two-thirds of EOC cases are diagnosed at an advanced stage or are unstaged at diagnosis, and the 5-year relative survival rate for these patients is approximately 30% [2]. Therefore, improved methods of early detection and prevention of this disease should be a health care priority and must be based on a deeper understanding of the pathogenesis of the disease [3].

Recent investigations of the pathogenesis of EOC have established that hormones and reproductive status (e.g., oral contraceptive use, parity, and breastfeeding) are the predominant risk and protective factors for this disease [2, 4, 5]. Recently, a joint project conducted by the World Cancer Research Fund and the American Institute for Cancer Research provided inconsistent and limited evidence linking dietary factors to EOC [6]. Experimental studies have hypothesized that high dietary fat intake may expose the ovarian epithelium to high levels of endogenous estrogens, which may trigger the development of EOC through cell damage and proliferation [7–9]. In 1986, Rose et al [10] conducted an ecologic study that suggested that high intake of dietary fat, particularly animal-based fat, was positively associated with EOC mortality. In 2001, a meta-analysis that included 8 case-control studies reported that patients with the highest intake of total fat, saturated fatty acids (FAs), and animal-based fat had a significantly increased risk of EOC compared with patients with the lowest intakes (relative risks [RRs]: 1.24, 1.20, and 1.70, respectively) [11]. Subsequently, a pooled analysis of 12 cohort studies in 2006 reported an opposite conclusion that indicated that intakes of total fat, planted-based fat, animal-based fat, monounsaturated FAs, polyunsaturated FAs, and trans-unsaturated FAs were not associated with EOC risk [12]. However, the results of the Women's Health Initiative Dietary Modification randomized controlled trial showed that a low-fat diet was associated with a reduced risk of EOC compared to a normal diet [13]. Furthermore, evidence from several recent epidemiological studies has been conflicting [14–19]. To our knowledge, a comprehensive assessment of the relationships between intakes of specific types of fat (i.e. planted-based and animal-based fats and monounsaturated, polyunsaturated, and trans-unsaturated FAs) and the risk of EOC has not been reported. Therefore, we conducted this meta-analysis of epidemiological studies to systematically assess the evidence of associations between dietary fat and FA intakes with EOC risk.

## RESULTS

### Search results, study characteristics, and quality assessment

**Figure 1:**
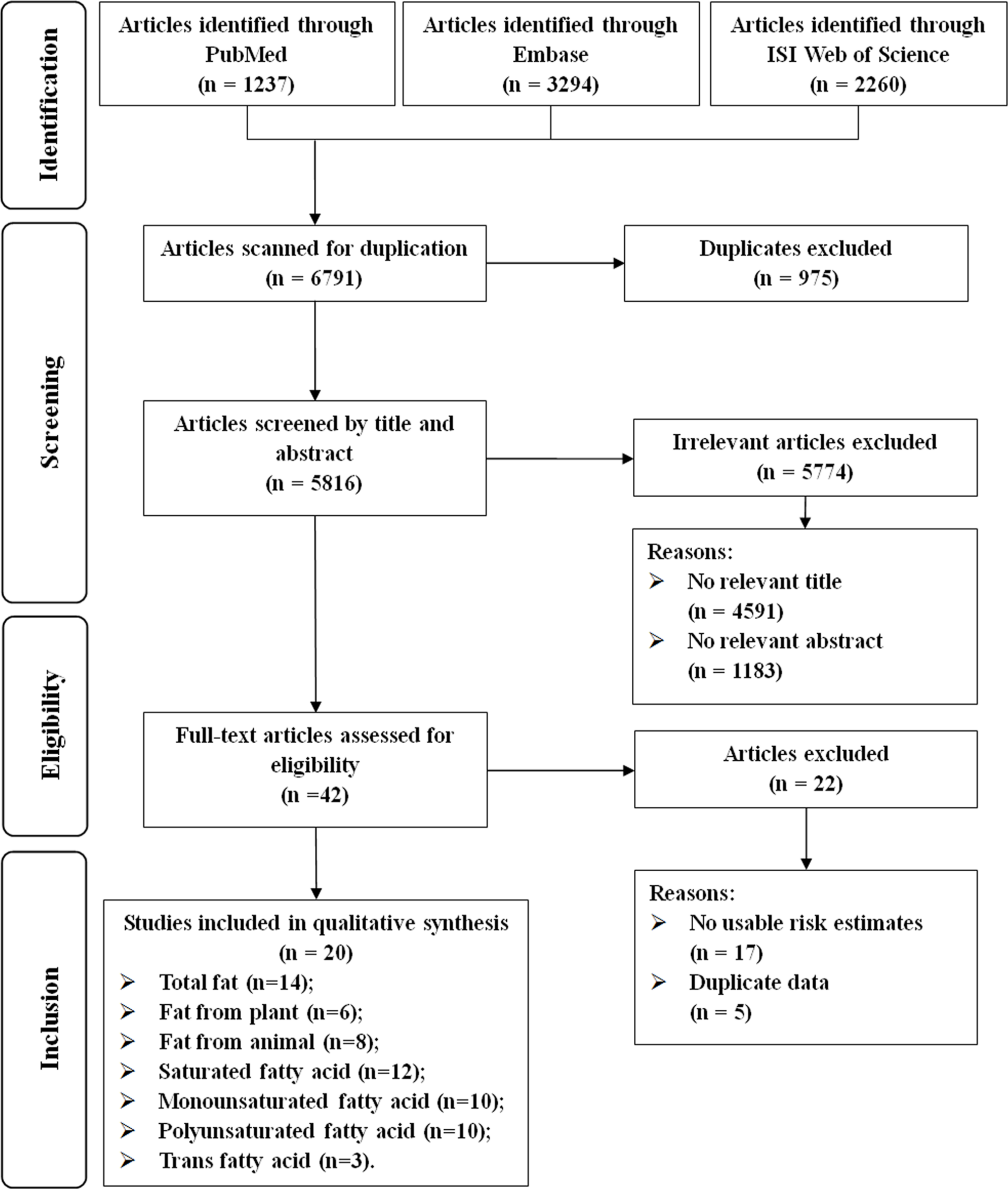
Flow-chart of study selection

Figure 1 illustrates the procedures we used to search and screen the articles. Briefly, the search strategy retrieved 6791 unique articles: 1340 from PubMed, 3294 from EMBASE, and 2260 from Web of Science. Of these, we excluded 6749 articles after the first screening on the basis of abstracts or titles. Among the 42 articles remaining for full-text review, 22 articles were excluded due to (i) a lack of reporting of risk estimates or 95% confidence intervals (CIs) and (ii) duplication of study populations. One pooled analysis included 12 cohort studies in the primary analyses [12], and we treated this pooled analysis as a single study in our meta-analysis. In all, we included 20 studies in our final analysis [12, 14–32].

The characteristics of the 20 selected studies are presented in Table 1. All of the studies were published between 1983 and 2014, and, together, they involved a total of 12,046 EOC cases and 1,105,946 non-cases. The 20 studies comprised 5 cohort studies, 14 case-control studies, and 1 pooled analysis. Of the 5 cohort studies, 3 were conducted in United States and 2 were conducted in Europe. Of the 14 case-control studies, 9 were conducted in North America, 3 were conducted in Europe, 1 was conducted in China, and 1 was conducted in Australia. Adjusted risk estimates were determined for all except for 2 of the studies [28, 32]. Most risk measures were adjusted for or stratified by age (18 studies), parity (15 studies), total energy intake (15 studies), and oral contraceptive use (12 studies); fewer were adjusted for menopausal status (9 studies), body mass index (5 studies), cigarette smoking status (5 studies), and alcohol drinking habits (4 studies).

**Table 1:** Characteristics of studies included in the meta-analysis

First author (ref), year, Country, Study name	No. of cases/cohort (controls)	Energy-adjusted model	Exposure categories(Dietary assessment)	Risk estimates (95% CI)	Matched/adjusted factors
**Prospective study**					
Merritt et al [14], 2014, Europe, EPIC	1191/325,007	Presented	**Quartile 4 *vs*. Quartile 1** Total dietary fat Plant-based fat Animal-based fat Saturated fatty acid Monounsaturated fatty acid Polyunsaturated fatty acid (Validated FFQ)	**Hazard ratio** 1.16 (0.96–1.40) 1.22 (0.98–1.52) 0.96 (0.80–1.15) 1.17 (0.97–1.40) 1.16 (0.93–1.44) 1.22 (1.02–1.48)	Ever use and duration of use of OC, number of children, menopausal status at enrolment, total energy intake and stratified by age and study center
Merritt et al [15], 2014, USA, NHS and NHSII	764/187,920	Presented	**Quintile 5 *vs*. Quintile 1** Dairy-based fat (Validated FFQ)	**Hazard ratio** 1.01 (0.80–1.27)	Total caloric intake, number of pregnancies and parity, OC use, menopausal status, tubal ligation, family history of ovarian cancer, and stratified by age in months, cohort, and time period
Blank et al [17], 2012, USA, NIH-AARP	695/151,552	Presented	**Quintile 5 *vs*. Quintile 1** Total dietary fat Plant-based fat Animal-based fat Saturated fatty acid Monounsaturated fatty acid Polyunsaturated fatty acid (Validated FFQ)	**Relative Risk** 1.28 (1.01–1.63) 1.00 (0.79–1.27) 1.30 (1.02–1.66) 1.03 (0.71–1.50) 1.01 (0.63–1.60) 1.28 (0.92–1.77)	Age, race, education, BMI, family history of ovarian cancer, duration of OC use, parity, duration of menopausal hormone therapy use, and total energy intake
Gilsing et al [19], 2011, Netherland, NCLS	340/62,573	Presented	**Quintile 5 *vs*. Quintile 1** Dairy-based fat (Validated FFQ)	**Relative Risk** 1.28 (0.91–1.80)	Age, total energy intake, parity, and use of OC
Genkinger et al [12], 2012, Multi-centers, AHS, BCDDP, CNBSS, CPSII, IWHS, NLCS, NYSC, NYU, NHS, NHSII, SMC, WHS	2132/523,217	Presented	**Quartile 4 *vs*. Quartile 1** Total dietary fat Plant-based fat Animal-based fat Saturated fatty acid Monounsaturated fatty acid Polyunsaturated fatty acid Trans unsaturated fatty acid (Validated FFQ)	**Relative Risk** 1.08 (0.94–1.24) 1.01 (0.87–1.18) 1.15 (0.99–1.33) 1.14 (0.97–1.34) 0.98 (0.86–1.12) 0.94 (0.80–1.09) 1.04 (0.84–1.28)	Age at menarche, menopausal status at baseline, OC use, hormone replacement therapy use among postmenopausal women, parity, BMI, smoking status, physical activity, and energy intake
Chang et al [20], 2007, USA, the California Teachers Study	280/97,275	Presented	**Quintile 5 *vs*. Quintile 1** Total dietary fat Saturated fatty acid (Validated FFQ)	**Relative Risk** 0.85 (0.58–1.24) 0.72 (0.48–1.08)	Race and total daily caloric intake, parity, use of OC, average strenuous PA, average daily consumption of alcohol from wine in the year before baseline, menopausal status/use of hormone therapy, and stratified by age at baseline
**Case-control study**					
Merritt et al [16], 2014, USA, PC-CS, NECC	1872/1978	Presented	**Quartile 4 *vs*. Quartile 1** Total dietary fat Plant-based fat Animal-based fat Saturated fatty acid Monounsaturated fatty acid Polyunsaturated fatty acid Dairy-based fat Trans unsaturated fatty acid (Validated FFQ)	**Odds Ratio** 1.07 (0.89–1.29) 0.98 (0.81–1.17) 1.04 (0.87–1.26) 1.11 (0.92–1.34) 0.97 (0.81–1.18) 0.82 (0.68–0.99) 0.95 (0.79–1.14) 1.30 (1.08–1.57)	Ever use and duration of use of OC, number of children, menopausal status at enrolment, total energy intake and stratified by age and study center
Hu et al [18], 2011, Canada, PC-CS, NECSS	442/5039	No	**Quartile 4 *vs*. Quartile 1** Trans unsaturated fatty acid (Validated FFQ)	**Odds Ratio** 1.04 (0.68–1.58)	Age, province, education, BMI, alcohol drinking, pack-year smoking, total of vegetable and fruit intake, monounsaturated fat, polyunsaturated fat, total energy intake, number of live births and years of menstruation
Pan et al [21], 2004, Canada, PC-CS, NECSS	442/2135	Considered	**Quartile 4 *vs*. Quartile 1** Total dietary fat Saturated fatty acid Monounsaturated fatty acid Polyunsaturated fatty acid (Validated FFQ)	**Odds Ratio** 1.21 (0.88–1.65) 1.06 (0.78–1.45) 1.26 (0.92–1.72) 1.28 (0.94–1.76)	Age, province of residence, education, alcohol consumption, cigarette pack-years, BMI, total caloric intake, recreational PA, number of live births, menstruation years, and menopause status
Bdoli et al [24], 2002, Italy, HC-CS, N/A	1031/2411	Considered	**Quintile 5 *vs*. Quintile 1** Total dietary fat Plant-based fat Animal-based fat Saturated fatty acid Monounsaturated fatty acid Polyunsaturated fatty acid (Validated FFQ)	0.60 (0.50–0.80) 0.60 (0.50–0.80) 0.90 (0.70–1.20) 0.80 (0.60–1.10) 0.70 (0.50–0.90) 0.70 (0.50–0.90)	Age, study center, year of interview, education, parity, OC use, and energy intake
McCann et al [22], 2002, USA, PC-CS, N/A	124/696	No	**Quartile 4 *vs*. Quartile 1** Total dietary fat Saturated fatty acid Monounsaturated fatty acid Polyunsaturated fatty acid (Validated FFQ)	**Odds Ratio** 1.51 (0.57–4.02) 1.46 (0.68–3.15) 1.77 (0.73–4.31) 0.63 (0.28–1.41)	Age, education, total months menstruating, difficulty becoming pregnant, OC use, menopausal status and total energy intake
Zhang et al [25], 2002, China, HC-CS, N/A	254/652	No	**Quartile 4 *vs*. Quartile 1** Animal-based fat (Validated FFQ)	**Odds Ratio** 4.55 (2.20–9.30)	Age, education, living area, BMI, smoking, alcohol drinking, tea drinking, family income, marital and menopause status, parity, tubal ligation, OC use, PA, family history of ovarian cancer, salted vegetables, preserved animal foods, fresh meat, fish and shellfish, poultry, eggs, milk and products, staple food, vegetables, fruits vegetable oil, and total energy intake
Salazar-Martinez et al [23], 2002, Mexico, HC-CS, N/A	84/629	Presented	**Tertile 3 *vs*. Tertile 1** Total dietary fat Plant-based fat Animal-based fat Saturated fatty acid Monounsaturated fatty acid Polyunsaturated fatty acid (Validated FFQ)	**Odds Ratio** 0.60 (0.33–1.06) 0.81 (0.46–1.45) 0.66 (0.37–1.19) 0.56 (0.31–1.02) 0.54 (0.30–0.99) 0.61 (0.34–1.11)	Age, total energy intake, number of live birth, recent changes in weight, physical activity and diabetes
Webb et al [26], 1998, Australia, PC-CS, N/A	824/1132	Considered	**Quartile 4 *vs*. Quartile 1** Total dietary fat (Validated FFQ)	**Odds Ratio** 1.86 (1.03–3.37)	Age group, education level, BMI, smoking, parity, OC use and total energy intake
Risch et al [27], 1994, Canada, PC-CS, N/A	450/564	No	**≥ 29.87 *vs*. < 19.17 g/d** Saturated fatty acid (FFQ)	**Odds Ratio** 1.38 (0.90–2.13)	Age at diagnosis, total calorie intake, number of full-term pregnancies, and total duration of OC use
Tzonou et al [28],* 1993, Greece, HC-CS, N/A	189/200	Considered	**≥ 110 *vs*. < 70 g/d** Total dietary fat ≥ 45 *vs*. < 25 g/d Saturated fatty acid **≥ 45 *vs*. < 25 g/d** Monounsaturated fatty acid **≥ 9 *vs*. < 5 g/d** Polyunsaturated fatty acid (FFQ)	**Odds Ratio** 0.98 (0.48–2.02) 0.80 (0.39–1.64) 0.45 (0.17–1.21) 0.78 (0.43–1.41)	N/A
Slattery et al [29], 1989, USA, PC-CS, N/A	85/492	No	**Tertile 3 *vs*. Tertile 1** Total dietary fat Saturated fatty acid Monounsaturated fatty acid Polyunsaturated fatty acid (Diet history)	**Odds Ratio** 1.30 (0.70–2.30) 1.30 (0.60–2.60) 1.30 (0.70–2.30) 1.20 (0.60–2.30)	Age, BMI, and number of pregnancy
La Vecchia et al [30], 1987, Italy, HC-CS, N/A	455/1385	No	**High *vs.* Low** Total dietary fat (FFQ)	**Odds Ratio** 2.14 (1.59–2.88)	Age
Cramer et al [31], 1984, USA, PC-CS, N/A	215/215	No	**≥ 225 *vs*. < 125 intake score** Animal-based fat (FFQ)	**Relative Risk** 1.83 (1.00–3.38)	Age, race, residence, and parity
Byers et al [32],* 1983, USA, HC-CS, N/A	274/1034	No	**Tertile 3 *vs*. Tertile 1** Total dietary fat (FFQ)	**Relative Risk** 1.25 (0.90–1.73)	N/A

AHS, Adventist Health Study; BCDDP, Breast Cancer Detection Demonstration Project Follow-up Study; BMI, body mass index; CI, confidence interval; CNBSS, Canadian National Breast Screening Study; CPSII, Cancer Prevention Study II Nutrition Cohort; EPIC, European Prospective Investigation into Cancer and Nutrition; FFQ, food frequency questionnaire; HC-CS, hospital-based case-control study; IWHS, Iowa Women's Health Study; NECC, New England Case-Control study; NECSS, National Enhanced Cancer Surveillance System; NYSC, New York State Cohort; NYU, New York University Women's Health Study; PC-CS, population-based case-control study; N/A, not available; NCLS, Netherlands Cohort Study; NHS, Nurses' Health Study; NHSII, Nurses' Health Study II; NIH-AARP, National Institutes of Health-American Association of Retired Persons; PA, physical activity; OC, oral contraceptive; SMC, Swedish Mammography Cohort; WHS, Women's Health Study.

* OR and 95% CI were calculated from published data with EpiCalc 2000 software (version 1.02; Brixton Health).

Information collected for the assessment of study quality is presented in Tables 2 and 3. Briefly, all cohort studies, except for 2, were assigned a star because they included a follow-up period that was long enough for outcomes to occur [17, 20]; for the 2 exceptions in this category, the mean follow-up period was less than 10 years (Table 2). Additionally, 6 case-control studies [23–25, 28, 30, 32] were not assigned a star in the selection of control subjects category because the controls included in the studies did not come from the same population as the cases. Nine case-control studies [16, 18, 21–27] were assigned 2 stars in the control for important factors or additional factors category because they adjusted for more than 2 important confounders in the multivariable analysis. In the exposure assessment category, 6 case-control studies [27–32] were not assigned a star because their food frequency questionnaires (FFQs) were not validated. Seven case-control studies [18, 22–24, 28, 30, 31] were assigned a star because there were no differences in response rates between cases and controls, and 6 case-control studies [16, 21, 23, 24, 26, 28] were assigned a star because there were no differences in presenting or considering energy-adjusted models in their primary analyses (Table 3). Compared with cohort studies, more case-control studies were below the threshold for quality assessment of observational studies recommended by the updated Newcastle-Ottawa Scale (NOS).

**Table 2:** Methodological quality of cohort studies included in the meta-analysis*

First author (reference), publication year	Representativeness of the exposed cohort	Selection of the unexposed cohort	Ascertainment of exposure	Outcome of interest not present at start of study	Control for important factor or additional factor^†^	Assessment of outcome	Follow-up long enough for outcomes to occur^‡^	Adequacy of follow-up of cohorts^§^	Using energy-adjusted model
Merritt et al [14], 2014	✯	✯	✯	✯	✯✯	✯	✯	✯	✯
Merritt et al [15], 2014	✯	✯	✯	✯	✯✯	✯	✯	✯	✯
Blank et al [17], 2012	✯	✯	✯	✯	✯✯	✯	—	✯	✯
Gilsing et al [19], 2011	✯	✯	✯	✯	✯✯	✯	✯	✯	✯
Genkinger et al [12], 2012	✯	✯	✯	✯	✯✯	✯	✯	✯	✯
Chang et al [20], 2007	✯	✯	✯	✯	✯✯	✯	—	✯	✯

* A study could be awarded a maximum of one star for each item except for the item Control for important factor or additional factor. The definition/explanation of each column of the Newcastle-Ottawa Scale is available from (http://www.ohri.ca/programs/clinical_epidemiology/oxford.asp.).

† A maximum of 2 stars could be awarded for this item. Studies that controlled for total energy intake received one star, whereas studies that controlled for other important confounders such as parity, oral contraceptive use received an additional star.

‡ A cohort study with a follow-up time > 10 y was assigned one star.

§ A cohort study with a follow-up rate > 75% was assigned one star.

**Table 3:** Methodological quality of case-control studies included in the meta-analysis*

First author (reference), publication year	Adequate definition of cases	Representativeness of cases	Selection of control subjects	Definition of control subjects	Control for important factor or additional factor^†^	Exposure assessment	Same method of ascertainment for all subjects	Non-response Rate^‡^	Using energy-adjusted model
Merritt et al [16], 2014	✯	✯	✯	✯	✯✯	✯	✯	—	✯
Hu et al [18], 2011	✯	✯	✯	✯	✯✯	✯	✯	✯	—
Pan et al [21], 2004	✯	✯	✯	✯	✯✯	✯	✯	—	✯
McCann et al [22], 2003	✯	✯	✯	✯	✯✯	✯	✯	✯	—
Bdoli et al [24], 2002	✯	✯	—	✯	✯✯	✯	✯	✯	✯
Zhang et al [25], 2002	✯	✯	—	✯	✯✯	✯	✯	—	—
Salazar-Martinez et al [23], 2002	✯	✯	—	✯	✯✯	✯	✯	✯	✯
Webb et al [26], 1998	✯	✯	✯	✯	✯✯	✯	✯	—	✯
Risch et al [27], 1994	✯	✯	✯	✯	✯✯	—	✯	—	—
Tzonou et al [28], 1993	✯	✯	—	✯	—	—	✯	✯	✯
Slattery et al [29], 1989	✯	✯	✯	✯	✯	—	✯	—	—
La Vecchia et al [30], 1987	✯	✯	—	✯	—	—	✯	✯	—
Cramer et al [31], 1984	✯	✯	✯	✯	✯	—	✯	✯	—
Byers et al [32], 1983	✯	✯	—	✯	—	—	✯	—	—

* A study could be awarded a maximum of one star for each item except for the item Control for important factor or additional factor. The definition/explanation of each column of the Newcastle-Ottawa Scale is available from (http://www.ohri.ca/programs/clinical_epidemiology/oxford.asp.).

† A maximum of 2 stars could be awarded for this item. Studies that controlled for total energy intake received one star, whereas studies that controlled for other important confounders such as parity, oral contraceptive use received an additional star.

‡ One star was assigned if there was no significant difference in the response rate between control subjects and cases by using the chi-square test (*P* > 0.05).

### Fat intake

**Figure 2:**
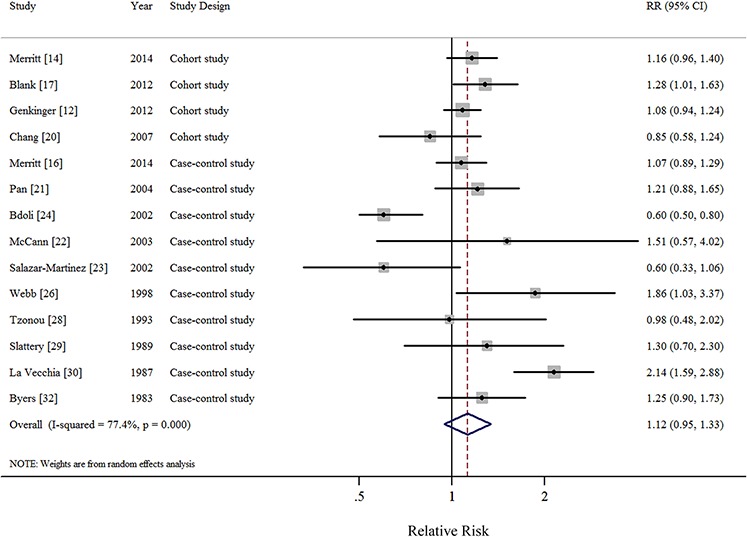
Forest plots (random effect model) of meta-analysis on the relationship between total dietary fat intake and epithelial ovarian cancer risk Squares indicate study-specific risk estimates (size of the square reflects the study-specific statistical weight); horizontal lines indicate 95% CIs; diamond indicates the summary relative risk with its 95% CI. RR: relative risk.

**Figure 3:**
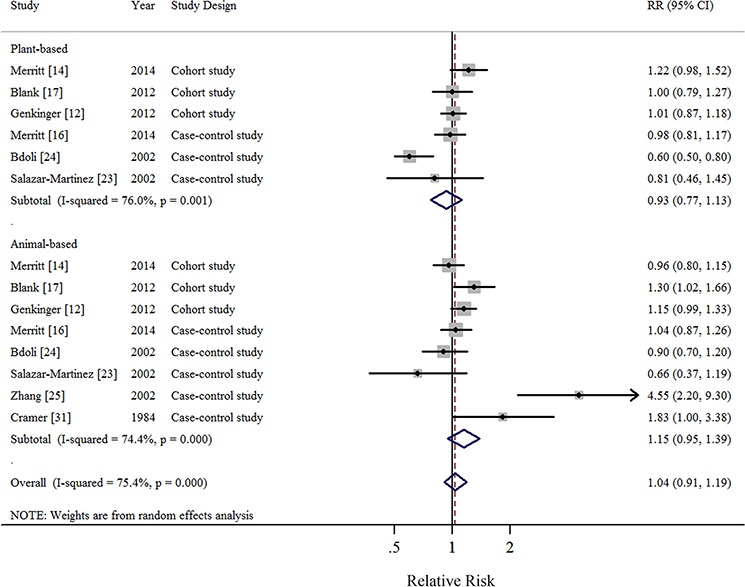
Forest plots (random effect model) of meta-analysis on the relationship between plant-based and animal-based fat intake and epithelial ovarian cancer risk Squares indicate study-specific risk estimates (size of the square reflects the study-specific statistical weight); horizontal lines indicate 95% CIs; diamond indicates the summary relative risk with its 95% CI. RR: relative risk.

**Figure 4:**
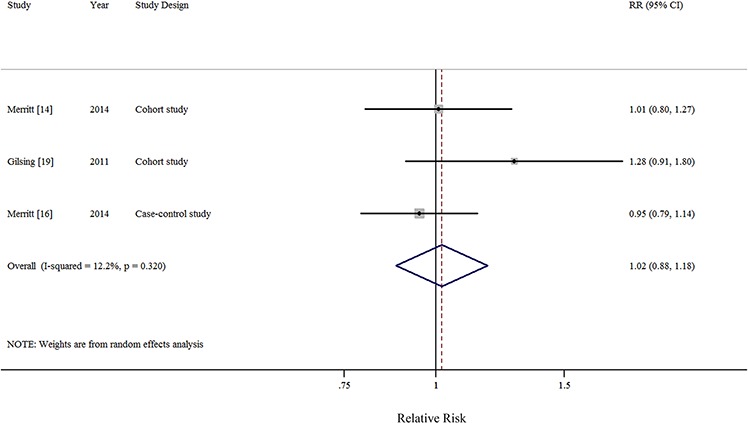
Forest plots (random effect model) of meta-analysis on the relationship between dairy-based fat intake and epithelial ovarian cancer risk Squares indicate study-specific risk estimates (size of the square reflects the study-specific statistical weight); horizontal lines indicate 95% CIs; diamond indicates the summary relative risk with its 95% CI. RR: relative risk.

**Figure 5:**
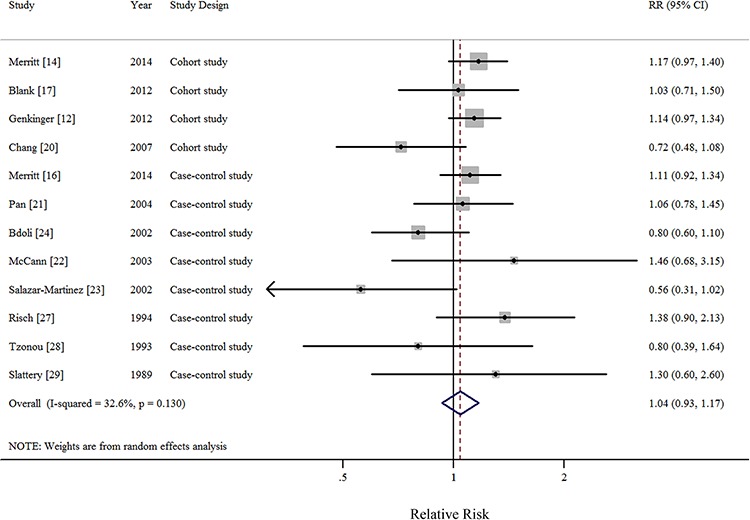
Forest plots (random effect model) of meta-analysis on the relationship between saturated fatty acid intake and epithelial ovarian cancer risk Squares indicate study-specific risk estimates (size of the square reflects the study-specific statistical weight); horizontal lines indicate 95% CIs; diamond indicates the summary relative risk with its 95% CI. RR: relative risk.

**Figure 6:**
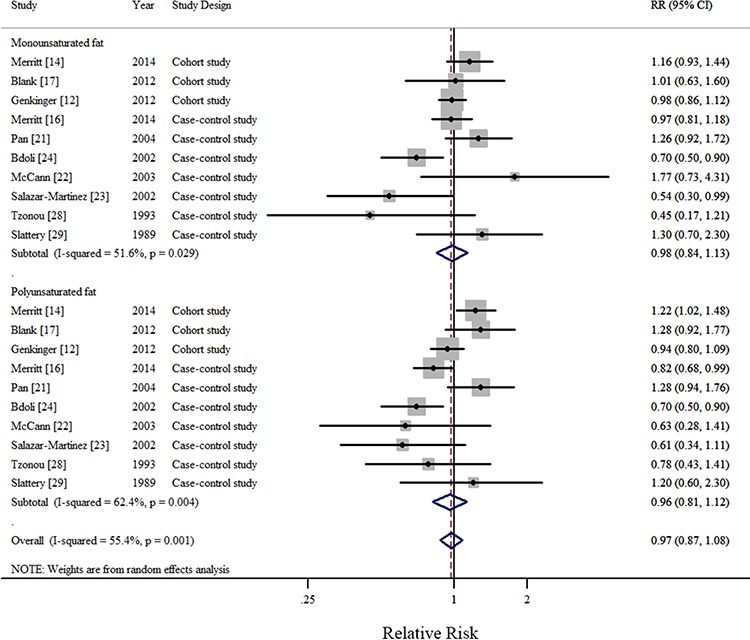
Forest plots (random effect model) of meta-analysis on the relationship between monounsaturated and polyunsaturated fatty acid intake and epithelial ovarian cancer risk Squares indicate study-specific risk estimates (size of the square reflects the study-specific statistical weight); horizontal lines indicate 95% CIs; diamond indicates the summary relative risk with its 95% CI. RR: relative risk.

**Figure 7:**
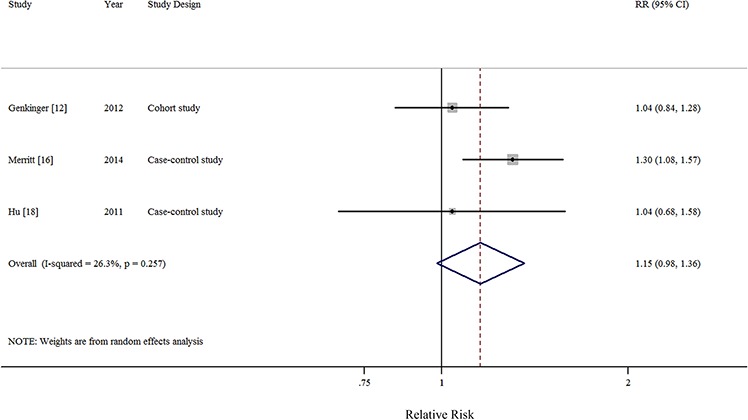
Forest plots (random effect model) of meta-analysis on the relationship between trans unsaturated fatty acid intake and epithelial ovarian cancer risk Squares indicate study-specific risk estimates (size of the square reflects the study-specific statistical weight); horizontal lines indicate 95% CIs; diamond indicates the summary relative risk with its 95% CI. RR: relative risk.

In all, 1 pooled analysis, 3 cohort, and 10 case-control studies investigated the relationship between total dietary fat intake and the risk of EOC. Comparison of the highest and lowest intake categories yielded a summary RR of 1.12 (95% CI = 0.95–1.33) with significant heterogeneity (*I*^2^ = 77.4%) (Figure 2). There was no indication of publication bias according to visual inspection of the funnel plot (Supplementary Figure S1) or by the Egger's test (*P* = 0.728). Non-significant results were also observed regarding intakes of plant-based fat (RR = 0.93, 95%CI = 0.77–1.13, *I*^2^ = 76.0%, *n* = 6) (Figure 3), animal-based fat (RR = 1.15, 95%CI = 0.95–1.39, *I*^2^ = 74.4%, *n* = 8) (Figure 3), dairy-based fat (RR = 1.02, 95%CI = 0.88–1.18, *I*^2^ = 12.2%, *n* = 3) (Figure 4), saturated FAs (RR = 1.04, 95%CI = 0.93–1.17, *I*^2^ = 32.6%, *n* = 12) (Table 5 and Figure 5), monounsaturated FAs (RR = 0.98, 95%CI = 0.84–1.13, *I*^2^ = 51.6%, *n* = 10) (Table 5 and Figure 6), polyunsaturated FAs (RR = 0.96, 95%CI = 0.81–1.12, *I*^2^ = 62.4%, *n* = 10) (Table 5 and Figure 6), and trans-unsaturated FAs (RR = 1.15, 95%CI = 0.98–1.36, *I*^2^ = 26.3%, *n* = 3) (Figure 7). The funnel plots of these associations are provided in Supplementary Figures S2 through S8.

### Subgroup and sensitivity analyses

Due to the limitations of the studies included in our meta-analysis, we only completed stratified analyses of intakes of total dietary fat, and saturated, monounsaturated, and polyunsaturated FAs across key study characteristics to explore study heterogeneity (Tables 4 and 5). Although several strata of the subgroup analyses revealed statistically significant associations, non-significant associations between total dietary fat intake and EOC risk were observed in the majority of the subgroup analyses stratified by type of control subjects, quality of study, geographic location, number of EOC cases, use of validated FFQ to collect dietary information or energy-adjusted model to analyze the associations between focused exposure and outcome, and adjustment for potential confounders (Table 4). Additionally, we detected no evidence of significant heterogeneity between subgroups on the basis of a meta-regression analysis. Similar patterns of no associations were observed in the subgroup analyses of dietary saturated, monounsaturated, and polyunsaturated FA intakes (Table 5).

**Table 4:** Summary risk estimates of the association between dietary fat intake and epithelial ovarian cancer risk (highest *versus* lowest)

	No. of study	Summary RR (95%CI)	*I*^2^ value (%)	*P*_h_*	*P*_h_**
**Total dietary fat**	14	1.12 (0.95–1.33)	77.4	< 0.001	
**Plant-based fat**	6	0.93 (0.77–1.13)	76.0	0.001	
**Animal-based fat**	8	1.15 (0.95–1.39)	74.4	< 0.001	
**Dairy-based fat**	3	1.02 (0.88–1.18)	12.2	0.320	
**Saturated fatty acid**	12	1.04 (0.93–1.17)	32.6	0.130	
**Monounsaturated fatty acid**	10	0.98 (0.84–1.13)	51.6	0.029	
**Polyunsaturated fatty acid**	10	0.96 (0.81–1.12)	62.4	0.004	
**Trans unsaturated fatty acid**	3	1.15 (0.98–1.36)	26.3	0.257	
**Subgroup analyses of total dietary fat**					
**Study design**					0.836
Cohort study	4	1.12 (1.00–1.25)	16.9	0.307	
Case-control study	10	1.15 (0.86–1.53)	83.2	< 0.001	
**Type of control subjects**					0.400
Population-based	5	1.16 (1.00–1.35)	0	0.455	
Hospital-based	5	1.00 (0.57–1.77)	91.7	< 0.001	
**Study quality**					0.116
High (≥8)	10	1.03 (0.87–1.23)	74.5	< 0.001	
Low (<8)	4	1.45 (1.01–2.08)	62.9	0.04	
**Geographic location**^†^					0.414
North America	4	1.10 (0.63–1.93)	93.3	< 0.001	
Europe	8	1.11 (0.97–1.28)	23.2	0.245	
Others	1	1.86 (1.03–3.36)	N/A	N/A	
**Validated FFQ**					0.116
Yes	10	1.03 (0.87–1.23)	74.5	< 0.001	
No	4	1.45 (1.01–2.08)	62.9	0.044	
**Number of cases**					0.558
≥ 450	7	1.18 (0.92–1.51)	88.0	< 0.001	
< 450	7	1.06 (0.87–1.30)	20.9	0.270	
**Energy-adjusted model**					0.072
Yes	10	1.02 (0.86–1.21)	74.1	< 0.001	
No	4	1.56 (1.12–2.18)	52.7	0.096	
**Adjustment for potential confounders**					
**Total energy intake**					0.116
Yes	10	1.03 (0.87–1.23)	74.5	< 0.001	
No	4	1.45 (1.01–2.08)	62.9	0.044	
**Body mass index**					0.438
Yes	3	1.22 (0.98–1.51)	51.1	0.130	
No	11	1.08 (0.85–1.36)	80.9	< 0.001	
**Parity**					0.087
Yes	9	1.02 (0.85–1.22)	76.9	< 0.001	
No	5	1.47 (1.07–2.01)	50.6	0.088	
**Oral contraceptive use**					0.444
Yes	8	1.05 (0.86–1.27)	77.4	< 0.001	
No	6	1.23 (0.88–1.70)	72.4	0.003	

CI, confidence interval; FFQ, food frequency questionnaire; N/A, not available.

* *P*-value for heterogeneity within each subgroup.

** *P*-value for heterogeneity between subgroups with meta-regression analysis.

† Analysis excluding the pooled analysis.

**Table 5:** Subgroup analyses of the association between dietary Saturated, monounsaturated, and polyunsaturated fatty acid intake and epithelial ovarian cancer risk (highest *versus* lowest)

	Saturated fatty acid					Monounsaturated fatty acid					Polyunsaturated fatty acid				
	No. of study	Summary RR (95%CI)	*I*^2^ value (%)	*P*_h_*	*P*_h_**	No. of study	Summary RR (95%CI)	*I*^2^ value (%)	*P*_h_*	*P*_h_**	No. of study	Summary RR (95%CI)	*I*^2^ value (%)	*P*_h_*	*P*_h_**
**Study design**					0.71					0.53					0.13
Cohort study	4	1.07 (0.92–1.26)	39.2	0.18		3	1.02 (0.92–1.14)	0	0.43		3	1.11 (0.90–1.36)	65.3	0.06	
Case-control study	8	1.01 (0.85–1.21)	35.0	0.15		7	0.92 (0.71–1.20)	62.3	0.01		7	0.85 (0.69–1.05)	46.4	0.08	
**Type of control subjects**					0.03					0.03					0.17
Population-based	5	1.14 (0.99–1.32)	0	0.82		4	1.11 (0.91–1.35)	19.8	0.29		4	0.97 (0.71–1.33)	58.0	0.07	
Hospital-based	3	0.75 (0.58–0.97)	0	0.57		3	0.65 (0.50–0.84)	0	0.56		3	0.70 (0.55–0.89)	0	0.85	
**Study quality**					0.46					0.92					0.99
High (≥8)	9	1.02 (0.90–1.16)	43.0	0.08		7	0.98 (0.84–1.14)	54.3	0.03		8	0.96 (0.80–1.14)	69.6	< 0.01	
Low (<8)	3	1.22 (0.87–1.69)	0	0.43		2	0.82 (0.29–2.31)	69.5	0.07		2	0.94 (0.60–1.47)	0	0.35	
**Geographic location**^†^					0.77					0.42					0.78
North America	3	0.96 (0.71–1.31)	59.8	0.08		3	0.82 (0.51–1.31)	79.1	0.01		3	0.90 (0.59–1.38)	81.2	0.01	
Europe	8	1.03 (0.87–1.22)	33.6	0.16		6	1.04 (0.83–1.30)	41.2	0.13		6	0.98 (0.75–1.26)	61.4	0.03	
**Validated FFQ**					0.46					0.92					0.99
Yes	9	1.02 (0.90–1.16)	43.0	0.08		8	0.98 (0.84–1.14)	54.3	0.03		8	0.96 (0.80–1.14)	69.6	< 0.01	
No	3	1.22 (0.87–1.69)	0	0.43		2	0.82 (0.29–2.31)	69.5	0.07		2	0.94 (0.60–1.47)	0	0.346	
**Number of cases**					0.16					0.78					0.84
≥ 450	6	1.10 (0.99–1.22)	17.2	0.30		5	0.97 (0.84–1.11)	45.5	0.12		5	0.96 (0.79–1.17)	75.8	< 0.01	
< 450	6	0.90 (0.69–1.17)	31.1	0.20		5	0.97 (0.62–1.53)	62.7	0.03		5	0.92 (0.65–1.28)	45.0	0.12	
**Energy-adjusted model**					0.14					0.22					0.88
Yes	9	1.01 (0.89–1.14)	42.6	0.08		8	0.95 (0.81–1.11)	56.4	0.03		8	0.96 (0.81–1.14)	68.8	0.01	
No	3	1.38 (0.99–1.92)	0	0.98		2	1.43 (0.87–2.35)	0	0.57		2	0.91 (0.48–1.69)	30.7	0.23	
**Adjustment for potential confounders**															
**Total energy intake**					0.92					0.92					0.99
Yes	10	1.04 (0.92–1.18)	41.6	0.08		8	0.98 (0.84–1.14)	54.3	0.032		8	0.96 (0.80–1.14)	69.6	< 0.01	
No	2	1.02 (0.61–1.70)	0	0.35		2	0.82 (0.29–2.31)	69.5	0.070		2	0.94 (0.60–1.47)	0	0.35	
**Body mass index**					0.59					0.93					0.47
Yes	2	1.12 (0.97–1.30)	0	0.63		2	0.98 (0.87–1.12)	0	0.903		2	1.06 (0.79–1.42)	64.2	0.01	
No	10	1.01 (0.87–1.18)	42	0.08		8	0.97 (0.78–1.20)	62.3	0.010		8	0.91 (0.73–1.13)	66.6	0.10	
**Parity**					0.74					0.65					0.63
Yes	9	1.03 (0.91–1.17)	45.9	0.06		7	0.97 (0.83–1.12)	56.1	0.034		7	0.97 (0.81–1.16)	72.6	< 0.01	
No	3	1.14 (0.74–1.74)	0	0.48		3	1.07 (0.53–2.16)	56.3	0.101		3	0.86 (0.58–1.27)	0	0.45	
**Oral contraceptive use**					0.42					0.89					0.87
Yes	8	1.07 (0.95–1.21)	34.7	0.15		6	0.98 (0.85–1.13)	44.8	0.107		6	0.95 (0.78–1.14)	71.6	< 0.01	
No	4	0.91 (0.65–1.27)	32.2	0.22		4	0.87 (0.53–1.45)	68.1	0.024		4	0.97 (0.67–1.39)	49.0	0.12	

CI, confidence interval; FFQ, food frequency questionnaire; N/A, not available.

* *P*-value for heterogeneity within each subgroup.

** *P*-value for heterogeneity between subgroups with meta-regression analysis.

† Analysis excluding the pooled analysis.

In a sensitivity analysis of total dietary fat intake and EOC risk, we sequentially removed 1 study at a time and re-analyzed the data. The 14 study-specific RRs ranged from a low of 1.06 (95%CI = 0.91–1.23, *I*^2^ = 67.5%, *P* < 0.001) after omitting the study by La Vecchia et al [30] to a high of 1.20 (95%CI = 1.04–1.37, *I*^2^ = 59.3%, *P* = 0.003) after omitting the study by Bdoli et al [24].

## DISCUSSION

Findings of this meta-analysis of 20 epidemiological studies suggest that total dietary fat intake may not be associated with an increased risk of EOC. When we investigated the associations according to fat source and FA type, we still observed non-significant results. These findings were robust in the majority of subgroup analyses according to study characteristics and sensitivity analyses.

Our findings are inconsistent with a previous meta-analysis of 8 case-control studies that reported that intakes of total dietary fat (RR = 1.24, 95%CI = 1.07–1.43), saturated FAs (RR = 1.20, 95%CI = 1.04–1.39), and animal-based fat (RR = 1.70, 95%CI = 1.43–2.03) were associated with an increased risk of EOC [11]. However, all 8 of the studies included in that analysis were retrospectively designed studies with inherent biases, including recall bias and selection bias; such biases may be avoided in cohort studies. Most of the 8 case-control studies were included in our current meta-analysis. However, after completing the quality assessment, we noted that more case-control studies than cohort studies were below the quality threshold recommended in the updated NOS. For example, 6 of 14 case-control studies used hospital-based controls, and differences in response rates were observed between cases and controls in half of these studies (Table 3). Still, our findings were largely in accordance with a previous pooled analysis of 12 cohort studies [12]. We excluded this pooled analysis and included 3 cohort studies in the sensitivity analysis and the results suggested that our findings were robust.

The present meta-analysis revealed no significant associations between intakes of dietary fat and FAs and EOC risk. Nevertheless, several plausible biological mechanisms have been proposed for the association between fat intake and EOC risk. Dietary fat has been hypothesized to affect ovarian carcinogenesis primarily through hormone-related mechanisms, which were well investigated by the researchers of previous studies. High dietary fat intake may expose the ovarian epithelium to high levels of endogenous estrogens, which may trigger the development of EOC through cell damage and proliferation [7–9]. Several previous studies observed lower urinary levels of total estrogens and estriol, higher fecal estrogen excretion, and higher levels of sex hormone-binding globulin in vegetarian women that consume low-fat diets compared to non-vegetarian women that consume normal diets that are higher in fat [33, 34]. These findings strongly support biological mechanisms of EOC development. In contrast, a cross-sectional study of 381 postmenopausal participants in the Nurses' Health Study found plasma estradiol levels were inversely related to intake of several specific fats [35]. Further experimental studies are needed to clarify the associations between specific dietary fat intake and the development of EOC.

Several recent studies have reported inconsistent associations between dietary fat and FA intakes and EOC risk across different histological subtypes and tumor invasiveness (borderline versus invasive) of EOC. For example, the European Prospective Investigation into Cancer and Nutrition (EPIC) found a borderline significant increased risk for serous EOC with a high intake of polyunsaturated FAs [14]. In contrast, Blank et al [17] observed a 10% increased risk of serous EOC per 5% increment of total energy from animal-based fat on the basis of the NIH-AARP Diet and Health Study. Furthermore, borderline significant inverse associations with risk of EOC were observed for the highest versus lowest quartiles of intakes of plant-based fat (odds ratio [OR] = 0.71) and polyunsaturated FAs (OR = 0.56); no significant associations were observed related to invasive EOC in a New England case-control study that included 1872 cases and 1978 population-based controls [16]. However, little evidence was observed for different types and sources of fat and FA intake between serous, endometrioid, and mucinous subtypes of EOC in the pooled analysis. Limited studies have demonstrated these results, so more studies should investigate and report the associations between fat intake and EOC risk that are stratified by cancer grade and histological subtype of EOC.

The difference in point estimates of risk between North American and European populations might be attributed to differences in amounts of FA consumption. Merritt et al [16] reported that top quartiles of saturated, polyunsaturated, and monounsaturated FAs in an American population were equivalent to consumption of 24.4, 13.5, and 25.5 g/day, respectively, in the New England case-control study. On the basis of the EPIC investigation, Merritt et al [14] reported that the top quartiles of consumption of saturated, polyunsaturated, and monounsaturated FAs in a European population equated to 17.2, 8.2, and 16.5 g/day, respectively, [14].

The present meta-analysis had several strengths. This is the most up-to-date meta-analysis available and it comprised systematic searching, detailed heterogeneity analysis, and study quality evaluations. Additionally, large numbers of EOC cases and non-cases were included, which increased the statistical power to identify clinically meaningful associations. The negative findings were robust in numerous subgroup and sensitivity analyses. However, several potential limitations should be considered. First, although all studies included in this meta-analysis were adjusted for multiple potential confounders, with the exception of 2 studies that provided crude risk estimates calculated from raw data, the possibility of residual confounding by imprecisely or unmeasured factors cannot be excluded. This issue may partly explain the difference between the findings of the Women's Health Initiative Dietary Modification randomized controlled trial and the conclusions of our present meta-analysis, since the former study specifically provided evidence of associations between a low-fat dietary pattern and postmenopausal EOC risk. Therefore, further studies are warranted to rule out residual confounding factors and confirm our findings. Second, the range of dietary fat intake may be underestimated and the magnitude of the associations between intake and risk of cancer may be overestimated because of possible misclassification of fat and FA intakes [36]. However, none of the studies included in our analysis provided risk estimates that were corrected for measurement errors, which could introduce limitations. Further, using only self-reported dietary assessment instead of biological markers to calculate dietary fat and FA exposures might limit the interpretation of results. However, stratified analyses indicated that using a validated FFQ did not significantly change the observed associations (Tables 2 and 3). Further, the dietary information collected in the cohort studies was based on a single assessment at baseline, and we were unable to assess and account for changes in dietary fat intake and food compositions over time. Third, significant heterogeneity was present among the studies, which indicates that considerable variability existed in the data. Stratified analyses were conducted to address the contribution of potential sources of clinical heterogeneity, such as study design, geographic location, number of EOC cases, and adjustment for potential confounders. Although the results of a meta-regression analysis indicated that these characteristics might not be the source of heterogeneity, the heterogeneity remained unexplained in several subgroups (Tables 2 and 3). These issues might also be attributed to the limited number of included studies. Finally, polyunsaturated FA includes several different FAs, such as omega-6 and omega-3 FAs, which might be associated with EOC risk. However, only a limited number of studies included in our analysis provided risk estimates of associations with these FAs and further studies are needed to investigate these associations.

In summary, the findings of the present meta-analysis, which included 1 pooled analysis, 5 cohort studies, and 14 case-control studies, provide limited evidence of an association between dietary fat intake, including total fat, plant-based fat, animal-based fat, dairy-based fat, and saturated, monounsaturated, polyunsaturated, and trans-unsaturated FAs, and EOC risk. Further prospective studies are needed to confirm the associations between specific types of fat and EOC risk, and the results should be stratified by tumor invasiveness and EOC histology.

## MATERIALS AND METHODS

### Search strategy

Two independent investigators (RH and Q-JW) systematically searched PubMed (MEDLINE), EMBASE, and Web of Science databases from each database's inception to the end of May 2015 to identify relevant epidemiological studies. The following keywords were used in the search: (diet OR dietary OR fat OR fatty) AND (ovarian OR ovary) AND (cancer OR tumor OR carcinoma OR neoplasm). Investigators also performed a manual review of references from eligible studies and several review articles [11, 37]. This search strategy was similar to that conducted in our previous studies [5, 38]. We followed the Preferred Reporting Items for Systematic Reviews and Meta-analyses (PRISMA) guidelines to plan, conduct, and report this meta-analysis [39].

### Study selection and exclusion

To be included in this analysis, a study must have (i) been an observational study design; (ii) evaluated the association between dietary fat and FA intakes and EOC risk; and (iii) presented RR, OR, or hazard ratio (HR) estimates with 95% CIs or data necessary for calculation of these risk estimates. Several cohort studies [19, 40, 41] published related literature before the pooled analysis of cohort studies [12] and we excluded results from earlier publications from this meta-analysis. If several publications involved overlapping individuals or populations, we included the study with more patients.

Studies were excluded if they (i) were randomized controlled trials, retrospective studies, reviews without original data, ecological studies, editorials, or case reports; (ii) reported risk estimates that could not be summarized (such as risk estimates without 95% CIs); and (iii) reported outcomes as EOC mortality or recurrence.

### Data abstraction and quality assessment

Data were extracted by one investigator (Q-JW) using a data extraction form and then entered into a database. An independent investigator (T-TG) checked the data and all differences were resolved by a third investigator (RH). For each included study, we extracted the following information: last name of the first author, publication year, geographic location, number of cases/controls (size of cohort), exposure assessment and categories, and study-specific adjusted estimates with 95% CIs (including information regarding adjusted confounders, if applicable). If there were multiple estimates for the associations, we used the estimate adjusted for the most appropriate confounding variables, as in previous studies [5, 42–44]. In situations when only unadjusted estimates were given, we used the unadjusted estimates.

The updated NOS [5, 42–46] recommends 4 quality parameters to assess the methodological quality of the studies included in our analysis: selection, comparability, exposure/outcome, and energy-adjusted model. We used these NOS parameters to evaluate the studies instead of scoring them and categorizing them into high- or low-quality on the basis of the scores; quality scoring might conceal important information by combining disparate study features into a single score and introduce an arbitrary subjective element into the analysis [47–49].

### Statistical analysis

Two studies [15, 19] presented risk estimates between dairy fat and FA intakes and EOC risk that were not provided in the pooled analysis [12]; we included these in the analysis of dairy fat. However, when summing the number of EOC cases and non-cases, we excluded these 2 studies [15, 19].

Similar to our previous studies [5, 38], we reported all results for this meta-analysis as RRs because of the low absolute risk of EOC and because the estimates of ORs from case-control studies and estimates of risk, rate, and HRs from cohort studies were all assumed to be valid estimates of the RR. We used the *I*^2^ metric to evaluate the between-study heterogeneity. *I*^2^ represents the ratio of between-study variance over the sum of the within-study and between-study variances and ranges from 0% and 100% [50]. We used random-effects models to estimate the summary RR for the associations between dietary fat and FA intakes and the risk of EOC [51]. Pre-specified subgroup analyses were conducted by study design (cohort *versus* case-control studies), type of control subjects (population-based *versus* hospital-based), geographic location (North America, Europe, and others), validated FFQ (yes *versus* no), number of EOC cases (≥ 450 *versus* < 450), energy-adjusted model (yes *versus* no), and adjustment for potential confounders including total energy intake, body mass index, parity, and oral contraceptive use. Heterogeneity between subgroups was evaluated by meta-regression analysis [5, 38, 42, 44, 46].

Small study bias, such as publication bias, can reflect genuine heterogeneity, chance, or other reasons for differences between small and large studies; we evaluated study bias with Egger's regression asymmetry test [52]. A *P*-value of 0.05 was used to determine whether significant publication bias existed. We also conducted sensitivity analyses by deleting each study in turn to reflect the influence of individual data on the overall estimate. All statistical analyses were performed with Stata software (version 12; StataCorp, College Station, TX).

## SUPPLEMENTARY FIGURES AND TABLES


