# Supplementary material for: Dietary fat and fatty acid intake and epithelial ovarian cancer risk: evidence from epidemiological studies
Source: Oncotarget. 2015 Oct 26;6(40):43099–119. doi: 10.18632/oncotarget.5525 (PMC4767494; doi:10.18632/oncotarget.5525)
Supplement: Supplementary file 1 [file oncotarget-06-43099-s001.pdf]

SUPPLEMENTARY FIGURES

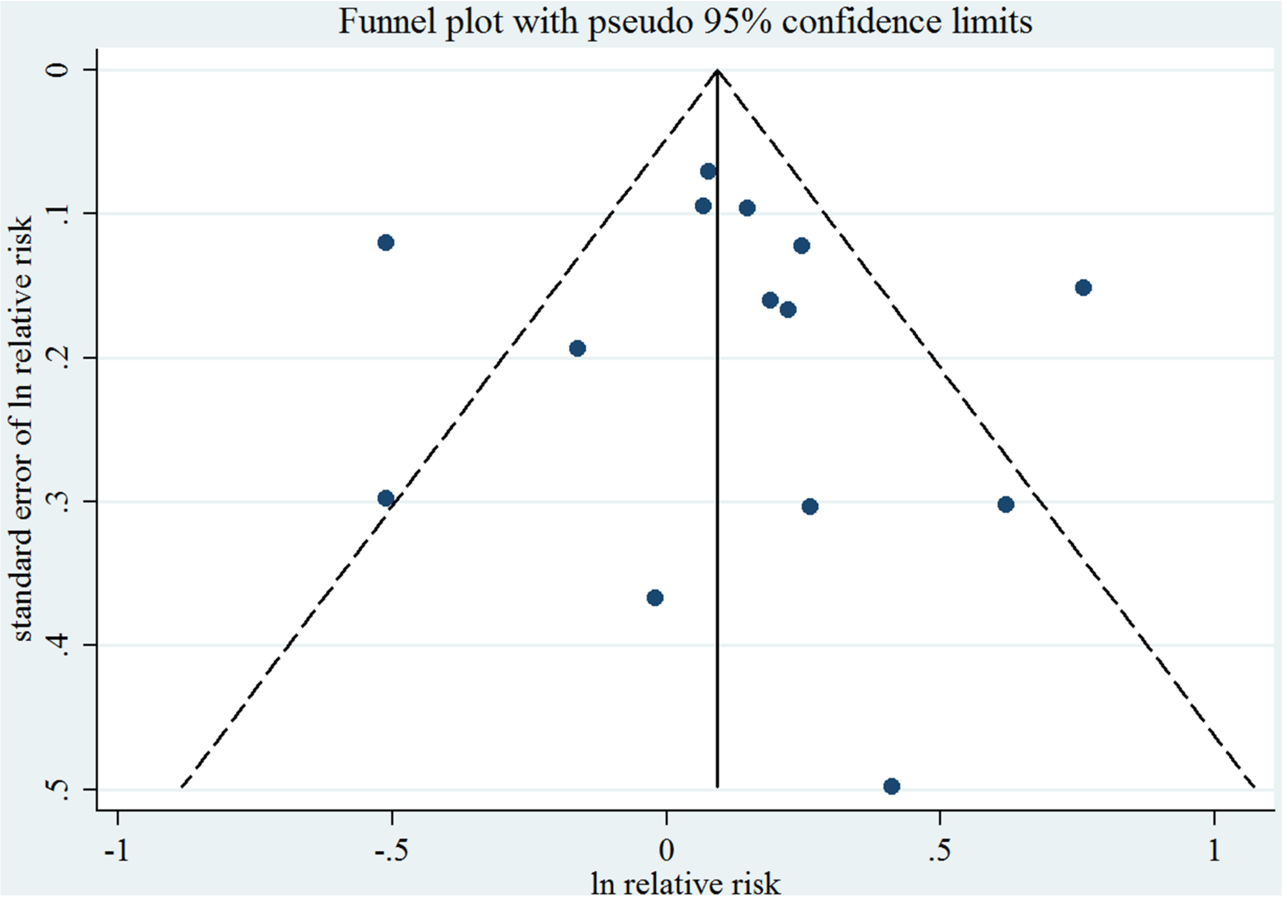

Supplementary Figure S1: Funnel plot corresponding to the random-effects meta-analysis of the relationship between total dietary fat intake and epithelial ovarian cancer risk.

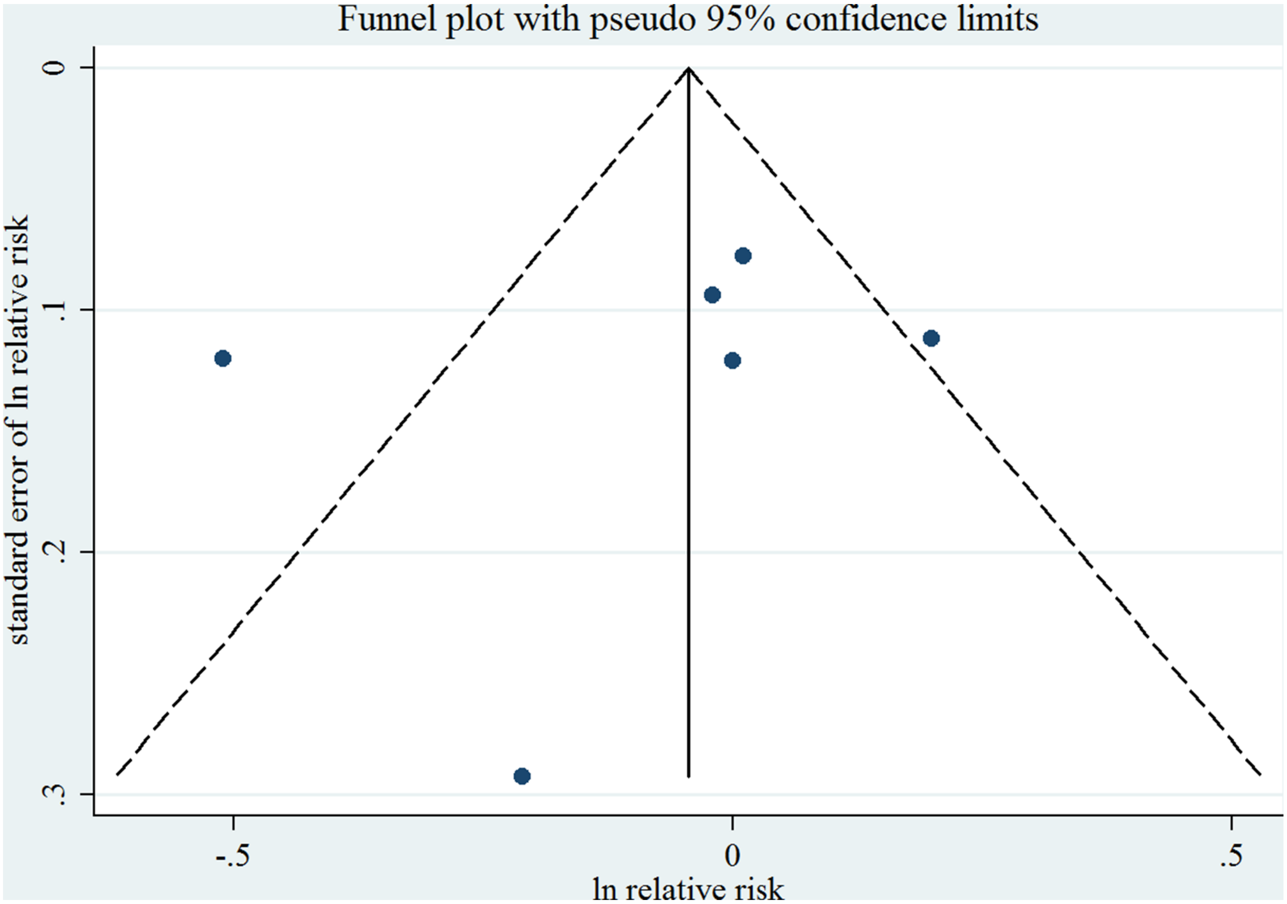

Supplementary Figure S2: Funnel plot corresponding to the random-effects meta-analysis of the relationship between plant-based fat intake and epithelial ovarian cancer risk.

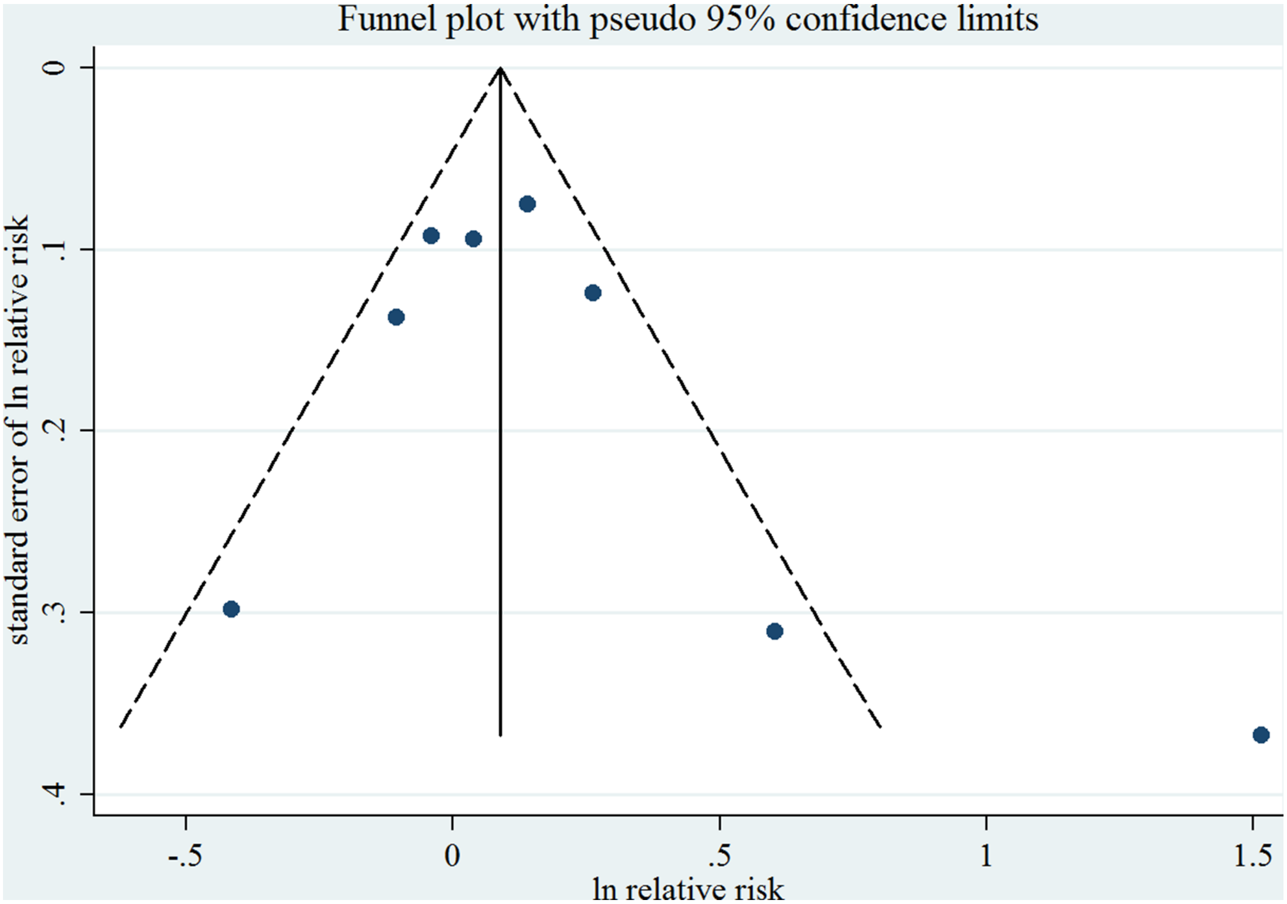

Supplementary Figure S3: Funnel plot corresponding to the random-effects meta-analysis of the relationship between animal-based fat intake and epithelial ovarian cancer risk.

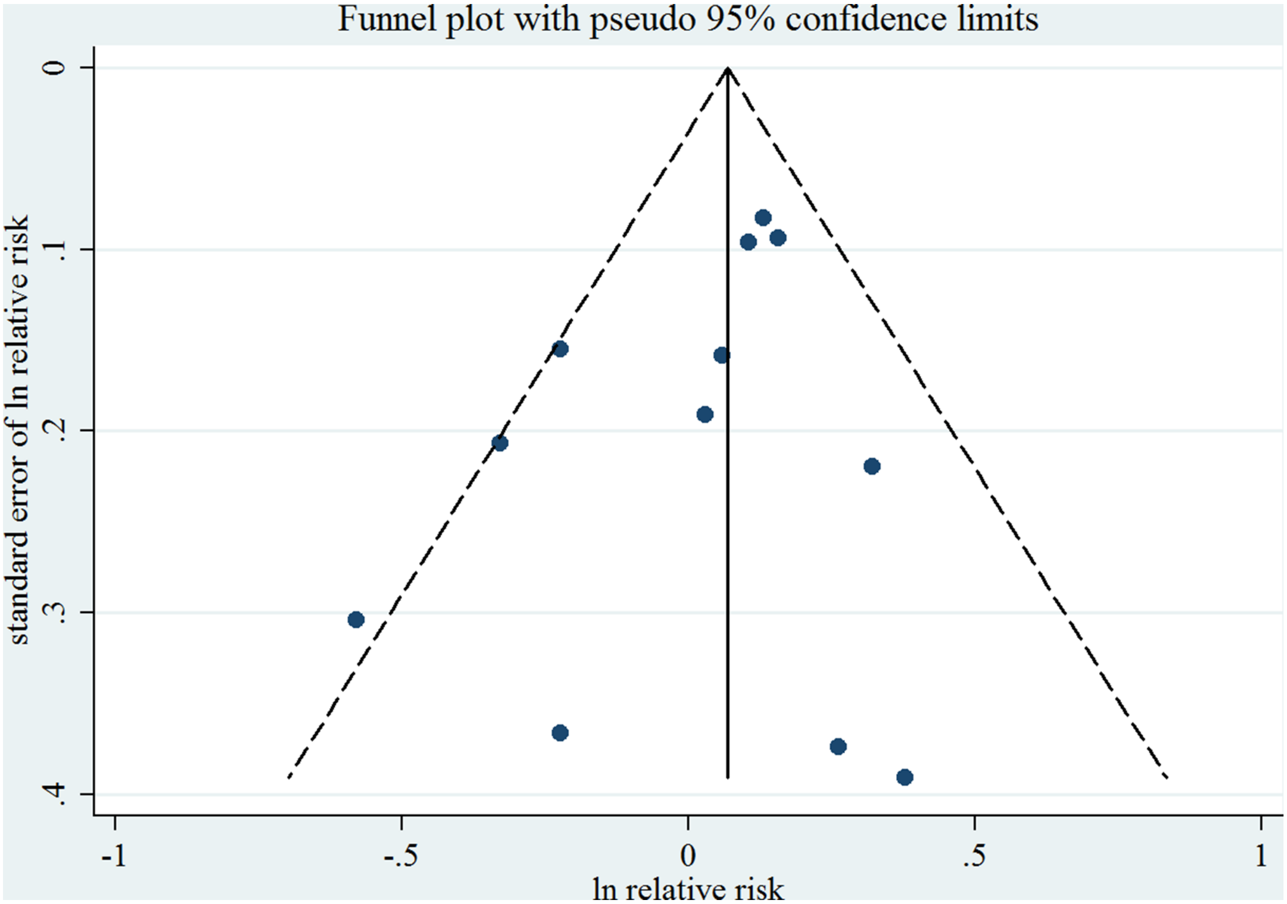

Supplementary Figure S4: Funnel plot corresponding to the random-effects meta-analysis of the relationship between saturated fatty acid intake and epithelial ovarian cancer risk.

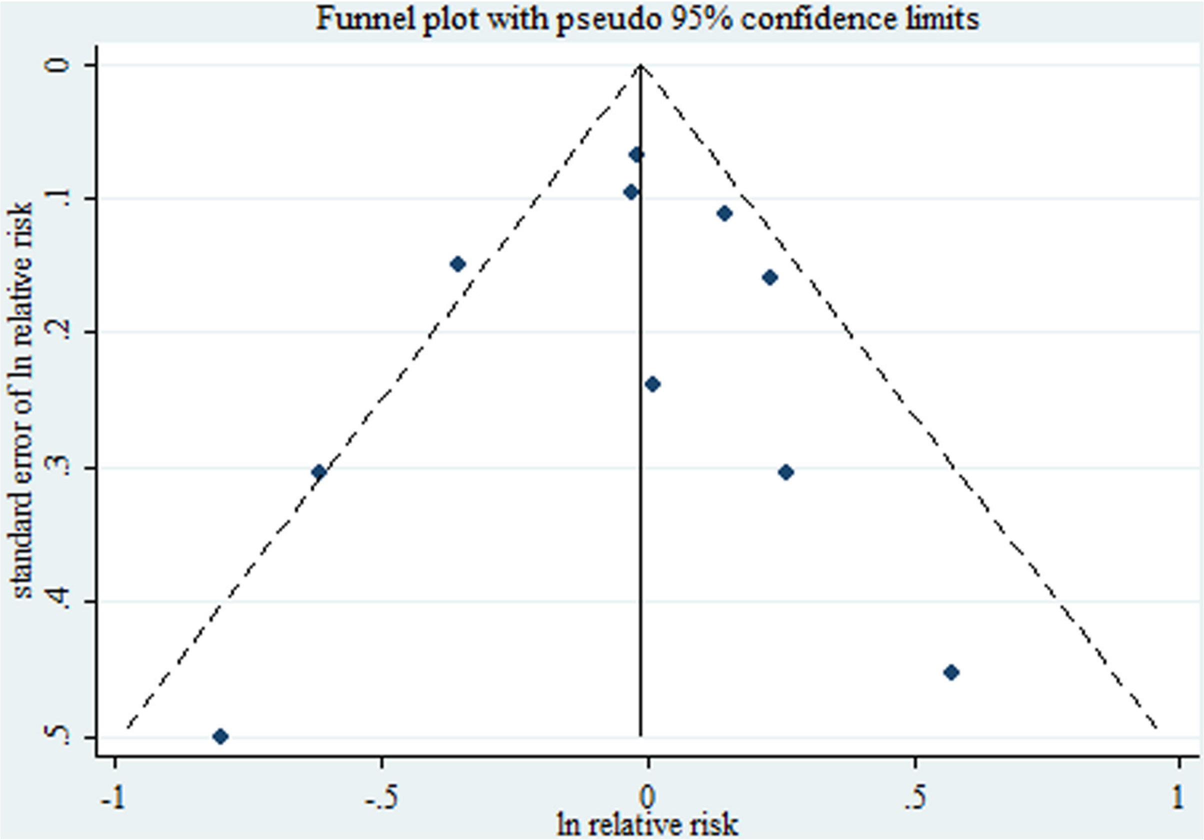

Supplementary Figure S5: Funnel plot corresponding to the random-effects meta-analysis of the relationship between monounsaturated fatty acid intake and epithelial ovarian cancer risk.

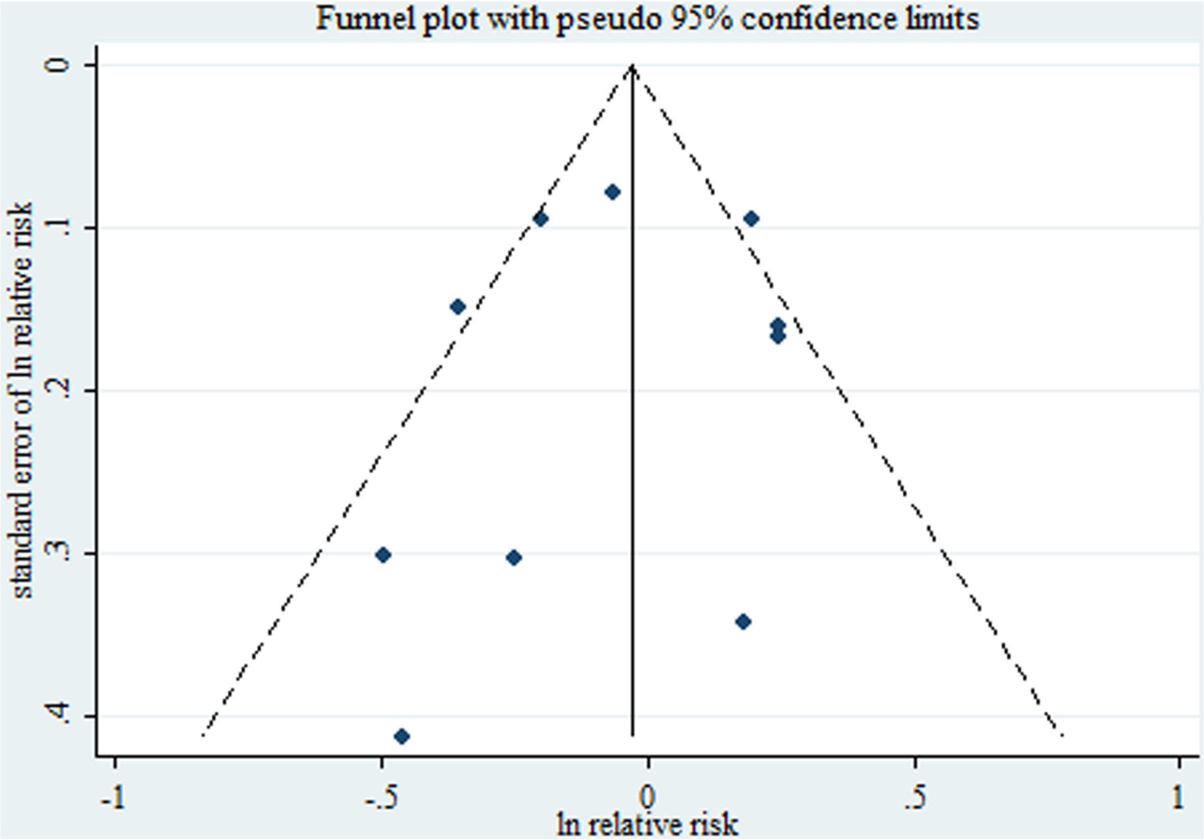

Supplementary Figure S6: Funnel plot corresponding to the random-effects meta-analysis of the relationship between polyunsaturated fatty acid intake and epithelial ovarian cancer risk.

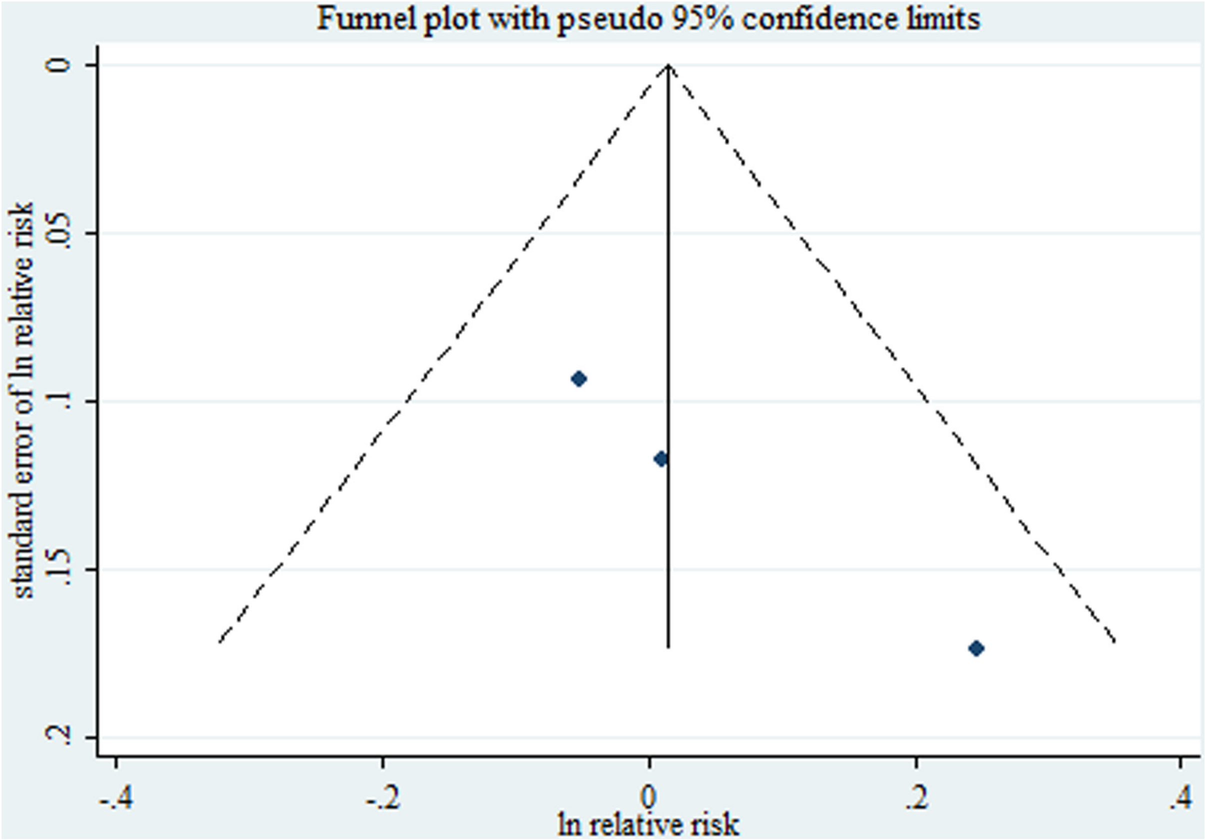

Supplementary Figure S7: Funnel plot corresponding to the random-effects meta-analysis of the relationship between dairy fat intake and epithelial ovarian cancer risk.

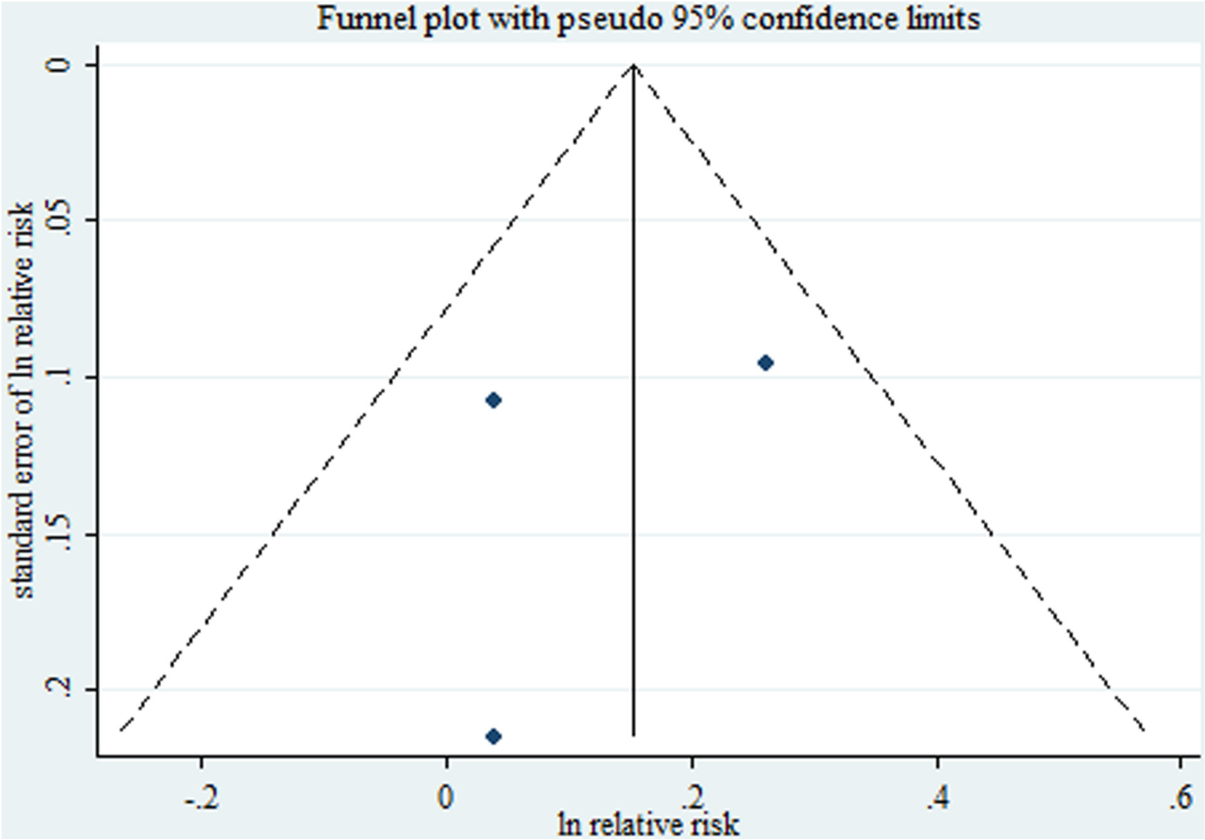

Supplementary Figure S8: Funnel plot corresponding to the random-effects meta-analysis of the relationship between trans unsaturated fatty acid intake and epithelial ovarian cancer risk.
